# Coagulation modifiers targeting SARS-CoV-2 main protease Mpro for COVID-19 treatment: an *in silico* approach

**DOI:** 10.1590/0074-02760200179

**Published:** 2020-06-01

**Authors:** Ísis Venturi Biembengut, Tatiana de Arruda Campos Brasil de Souza

**Affiliations:** 1 Fundação Oswaldo Cruz-Fiocruz, Instituto Carlos Chagas, Laboratório de Proteômica Estrutural e Computacional, Curitiba, PR, Brasil

**Keywords:** structure, ligand binding, *in silico* approach, COVID-19, SARS-CoV-2, Mpro

## Abstract

Severe acute respiratory syndrome coronavirus 2 (SARS-CoV-2) infection depends on viral polyprotein processing, catalysed by the main proteinase (Mpro). The solution of the SARS-CoV-2 Mpro structure allowed the investigation of potential inhibitors. This work aims to provide first evidences of the applicability of commercially approved drugs to treat coronavirus disease-19 (COVID-19). We screened 4,334 compounds to found potential inhibitors of SARS-CoV-2 replication using an *in silico* approach. Our results evidenced the potential use of coagulation modifiers in COVID-19 treatment due to the structural similarity of SARS-CoV-2 Mpro and human coagulation factors thrombin and Factor Xa. Further *in vitro* and *in vivo* analysis are needed to corroborate these results.

Humanity is trying to understand coronavirus disease-19 (COVID-19) at the same time severe acute respiratory syndrome coronavirus 2 (SARS-CoV-2) infection overcomes frontiers and spreads out through the world. Four human infecting species of coronavirus (HcoV-229E, HcoV-OC43, HcoV-NL63, and HcqV-HKU1) were already known as causative agent of cold and pneumonia; this viral family was responsible for the SARS-CoV and Middle East respiratory syndrome (MERS-CoV) outbreaks. The consequences from the species that emerged from Wuhan, China are far more catastrophic.

SARS-CoV-2 infection depends on viral polyprotein processing, an event catalysed by the main proteinase (Mpro) (also known as 3CLpro). Since Mpro is unique in the virus and not found in the host cells, this protein is a prominent target for the development of antivirals against coronavirus infections.^1^ Recently, the solution of the SARS-CoV-2 Mpro tridimensional structure allowed the investigation of potential inhibitors of viral replication. Mpro is composed of three domains (I- chymotrypsin, II- picornavirus 3C protease-like and III-globular cluster involved in protein dimerisation) and crystallography structures indicate the region between domains I and II for protein activity inhibition. That region contains the catalytic cysteine 145.

The crystal structure of SARS-CoV-2 Mpro was published by Zhang et al.^2^ We selected a list of existing antivirals drugs (64 compounds) and protease inhibitors (80 compounds) from the Drugbank Database^3^ as ligands. The DrugBank database was used for a larger screening, evaluating 4190 compounds randomly selected. The crystal protein structure (PDB 6Y2E) was energy-minimised using MMTK package on Chimera version 1.14.^4^ *In silico* molecular docking of ligand selection was performed using PyRx-virtual screening tool consisting of AutoDock^5^ and AutoDockvina.^6^

The PyRx-virtual screening tool was used for docking with the protocol: (i) the SARS-CoV-2 Mpro protein structure (PDB 6Y2E) was checked for missing atoms, bonds and contacts, removal of water molecules and energy minimisation was done with following parameters, force field: Amber ff14SB, steepest descent steps: 100, steepest descent step size: 0.02 Å, conjugate gradient steps: 10, conjugate gradient step size: 0.02 Å using molecular modelling toolkit (MMTK) package on Chimera version 1.14.^4^ This minimised structure was used as the receptor for docking analysis. (ii) The minimised structure was saved as a pdb file and imported into PyRx software. (iii) Ligands are imported in pdb format as well. Autodock Tools module was used to generate pdbqt input files. (iv) Autodock Vina algorithm was used to perform docking with the selected ligands. In Autodock Vina the grid box was set to cover the active site of Mpro with the following dimensions in Å: centre (x, y, z) = (-16.46, -26.70, 1.58), dimensions (x, y, z) = (23.34, 19.09, 10.98). The docking simulation was then run at an exhaustiveness of eight. The docking results were evaluated using the lowest Binding Affinity score (kcal/mol) predicted by the build in scoring function of Autodock Vina module.

The results showed 1,321 molecules with scores above 6.5 as Mpro partners [Supplementary data (Table)]. Due to the urgency in providing therapeutics to patients, we focus on the analysis of commercially approved drugs. Argatroban (blood clotting), Linagliptin (diabetes), Saquinavir (antiviral), Edoxaban (blood clotting), Apixaban (blood clotting), Cilazapril (ACE inhibitor), Betrixaban (blood clotting), Alogliptin (dipeptidyl peptidase 4 inhibitors), Sitagliptin (dipeptidyl peptidase 4 inhibitors), Ramipril (ACE inhibitor), Lopinavir (antiviral), Saxagliptin (diabetes), Indinavir (antiviral), Zofenopril (ACE inhibitor), Nelfinavir (antiviral), Quinapril (ACE inhibitor), Dihydroergotamine (antimigraine agents), Risperidone (atypical antipsychotics), Astemizole (antihistamine) presented the higher scores (Fig. 1). The list includes antiviral components (as expected) and drugs classified as coagulation modifiers (4) and ACE inhibitors (4).

**Fig. 1:**
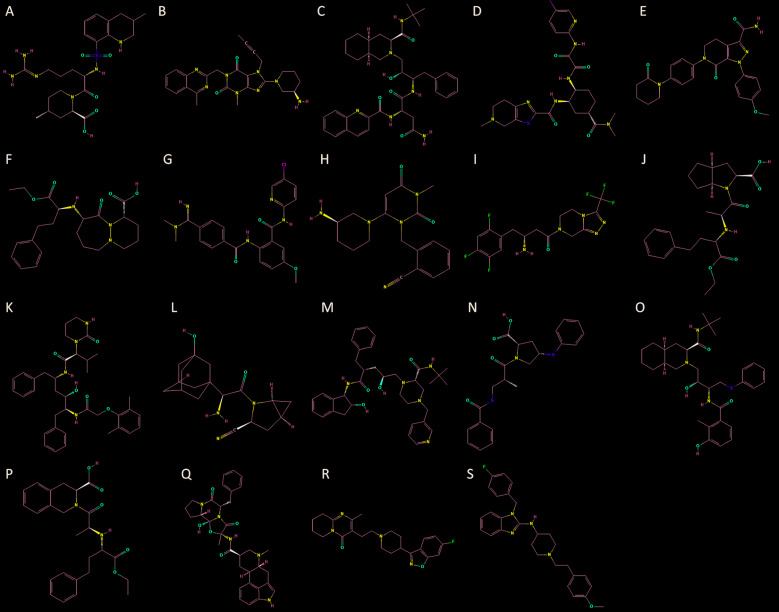
two dimensional representation of Argatroban (A), Linagliptin (B), Saquinavir (C), Edoxaban (D), Apixaban (E), Cilazapril (F), Betrixaban (H), Alogliptin (H), Sitagliptin (I), Ramipril (J), Lopinavir (K), Saxagliptin (L), Indinavir (M), Zofenopril (N), Nelfinavir (O), Quinapril (P), Dihydroergotamine (Q), Risperidone (R), Astemizole (S).

Lin et al.^7^ propose a pathogenic mechanism for the disease, based on clinical evidence and studies of other coronavirus strains. They divide the clinical phase in the viraemia phase, acute phase and the recovery. The specific effects of COVID-19 on the cardiovascular system remain unclear, though there have been reports of acute cardiac injury, arrhythmias, hypotension, tachycardia, and a high proportion of concomitant cardiovascular disease in infected individuals, particularly those who require more intensive care.^8^ Also, there are signs of coagulopathy in many patients, usually associated with death.^9^ Among coagulation modifiers, Argatroban is a direct thrombin inhibitor^10^ for prophylaxis or treatment of thrombosis in patients with heparin-induced thrombocytopenia. The three other compounds (Edoxaban, Apixaban, Betrixaban) bind to factor Xa.^11^ Edoxaban is used to prevent stroke and systemic embolism in patients with atrial fibrillation.^12^ Apixaban is indicated for the control of recurrence of thromboembolic events, such as stroke in patients with non-valve atrial fibrillation. Betrixaban inhibits free and prothrombinase bound factor Xa in a concentration-dependent manner.^13^ ^,^ ^14^ Betrixaban was more potent at inhibiting thrombin-antithrombin complex.^15^

**Fig. 2:**
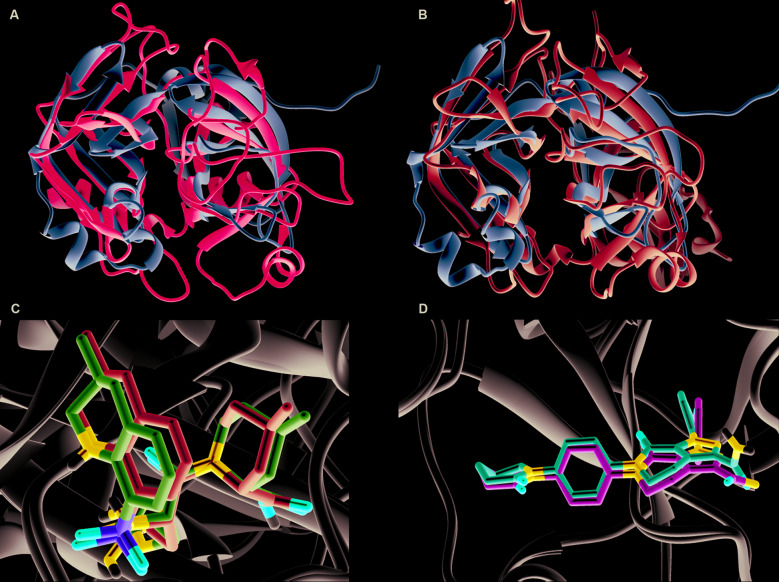
superposing of main proteinase (Mpro) (domains I and II) with thrombin (A) and factor Xa (B). C and D highlights the similarity of docked results and crystallography structures. C- Docked Argatroban (blue) compared to the experimentally solved (purple, PDB code 4HFP) structure complexed to thrombin. D- Apixaban docked with factor Xa (red) and its crystallography structure (green, PDB code 2P16).

Superposing of SARS-CoV-2 Mpro with factor Xa and thrombin structures presents a rmsd value of 2.57Ᾰ and 2.49Ᾰ, respectively. These low values are an indication of fold similarity (Fig. 2A-B). Thrombin, the known target of Argabotran, is a serine protease^16^ as Mpro is a protease itself with Leu-Gln↓(Ser,Ala,Gly) as a recognition sequence.^2^ This similarity may indicate a direct perturbation in pro-clotting coagulation by COVID-19 through Mpro protein. In fact, changes in pro-clotting factors in COVID-19 patients were already detected showing significant decreased antithrombin (AT) values and increased D-dimer, fibrin/fibrinogen degradation products (FDP) and fibrinogen (FIB).^17^ Additionally, the gradual progression of disease severity was mirrored by increasing values of D-dimer and FDP. This pattern might indicate the activation of a coagulation system due to thrombosis or disseminated intravascular coagulation (DIC).^18^ Heparin, as an anticoagulant therapy, was associated with a better prognosis in some patients.^9^ Also, the risk of developing DIC due to SARS-CoV-2 infection which has an obvious negative impact on the prognosis,^19^ ^,^ ^20^ was previously speculated.^21^ Moreover, excessive activation of fibrinolysis may be observed during cardiopulmonary bypass also leading to DIC and haemorrhage.^22^

The feasibility and robustness of our interactions are strengthened by comparing the docking results of thrombin interaction with Argatroban and the corresponding crystallographic structure deposited under the code 4HFP. Both structures present a rmsd of 0.49Ᾰ, indicating both docking and experimental structures are almost identical (Fig. 2C). In addition, the comparison of docking results of factor Xa interaction with Apixaban and the corresponding crystallographic structure deposited under the code 2P16 present a rmsd of 0.11Ᾰ, which also corroborates with the assertiveness of our approach (Fig. 2D). Finally, it is important to highlight that the score of thrombin interaction with Argatroban is 9.6 kcal/mol against 8.3 kcal/mol with SARS-CoV-2 Mpro. Otherwise, Apixaban interaction with Factor Xa presents a score of 5.1 kcal/mol against 7.0 kcal/mol with SARS-Cov-2 [Supplementary data (Table)]. All these are preliminary evidence that Argatroban has more affinity to Thrombin, but Apixaban tends to have more affinity to SARS-CoV-2 Mpro.

We also performed fold similarity investigation for the ACE inhibitors (ACEIs) with Mpro. The high rmsd values (6.03 Ᾰ and 5.57 Ᾰ for the ACE receptors structures deposited under the codes 2X92 and 2EWB, respectively) indicate the low structural conservation among those proteins. Also, the use of ACE inhibitors contributes for the up-regulation of ACE2 expression.^23^ ^,^ ^24^ Even as a potential target for the treatment of coronavirus infection, some groups suggest that the use of these inhibitors could lead to higher viral loads, because the ACE2 augmented expression.^25^ This could be a drawback for the use of ACEIs as COVID19 treatment.

In conclusion, we show the potential applicability of some coagulation compounds for the first time to our knowledge to treat COVID-19 infection. We highlight the fold similarity among SARS-CoV-2 Mpro and coagulation factors thrombin and Factor Xa. The effects of this similarity should urgently be investigated by *in vitro* approaches.
